# Molecular Mechanisms of Endocrine-Disrupting Chemicals and Emerging-Pollutant Toxicity in Human Reproduction: From Xenobiotic Exposure to Fertility Impairment and Reproductive Carcinogenesis

**DOI:** 10.3390/ijms27177766

**Published:** 2026-08-30

**Authors:** Zakhia El Beaino, Jean-Marc Ayoubi, Samir Hamamah

**Affiliations:** 1 Department of Pathology, Lariboisière Hospital APHP, 75010 Paris, France; 2 INSERM Unit 1203, Reproductive Biology & Environment, University of Montpellier, 34090 Montpellier, France; 3 Department of Obstetrics & Gynecology, Foch Hospital, 92150 Suresnes, France; jm.ayoubi@hopital-foch.com; 4 Institut de la Santé de la Femme et de la Fertilité, Faculty of Medicine, Université Versailles Saint-Quentin (UVSQ), 78000 Versailles, France; 5 Department of Obstetrics & Gynecology, Groupe Hospitalier Privé Ambroise Paré-Hartmann, 92200 Neuilly-sur-Seine, France

**Keywords:** oxidative stress, genotoxicity, microplastics, pharmaceuticals and personal-care products, hypothalamic–pituitary–gonadal axis, epigenetic reprogramming, phthalates, bisphenols, per- and polyfluoroalkyl substances, steroidogenesis

## Abstract

Human fertility is declining across industrialised populations, while the incidence of hormone-dependent reproductive cancers rises. Endocrine-disrupting chemicals (EDCs) and structurally related emerging pollutants are implicated in both. These two outcomes are generally reviewed as separate studies. This review argues that they are two latencies of a single molecular toxicology. The compounds concerned are structurally diverse: phthalates, bisphenols, per- and polyfluoroalkyl substances (PFASs), pesticides, polychlorinated biphenyls (PCBs) and dioxins, brominated and organophosphate flame retardants, pharmaceuticals and personal-care products (PPCPs), and micro- and nanoplastics. They nonetheless converge on a limited repertoire of molecular lesions. These include the disruption of hypothalamic–pituitary–gonadal (HPG) signalling through kisspeptin/GnRH and gonadotropin gene expression and interference at nuclear and membrane hormone receptors (ERα/β, AR, GPER, thyroid receptors, AhR, PPARγ). They also include the inhibition of steroidogenesis at StAR and the CYP11A1–CYP17A1–CYP19A1/3β-HSD/17β-HSD cascade and reactive-oxygen-species generation with mitochondrial dysfunction and Keap1–Nrf2 disruption. Epigenetic reprogramming through DNA methylation, histone modification and non-coding RNAs, together with crosstalk with metabolic and immune signalling, completes the set. These lesions produce measurable cytotoxic and genotoxic damage to gametes and the early embryo: sperm DNA fragmentation and 8-oxo-dG accumulation, blood–testis-barrier breakdown, oocyte meiotic-spindle defects, and granulosa-cell apoptosis and pyroptosis. The same receptor, oxidative and genotoxic hubs drive hormone-dependent reproductive carcinogenesis over longer latencies. The review makes three contributions. First, it traces these shared hubs continuously from fertility impairment to malignancy rather than treating them as separate fields. Second, it grades the certainty of the human evidence class by class, so that robust associations can be distinguished from provisional ones. Third, it integrates pseudo-persistent pollutants alongside the classical persistent compounds. These are micro- and nanoplastics, which act as both toxicants and vectors for adsorbed co-contaminants, and pharmaceutical and personal-care residues sustained by continuous wastewater input. Their inclusion demonstrates that chronic low-dose exposure does not require chemical persistence. We conclude with mitigation strategies and an explicit account of what the current evidence base cannot yet support.

## 1. Introduction

Infertility affects approximately one in six adults of reproductive age worldwide, and although its overall prevalence has remained relatively stable, converging evidence implicates the chemical environment as a modifiable contributor to declining human fecundity [1,2,3].

Classical explanations account for only part of the picture. In women, these include ovulatory dysfunction, tubal disease, endometriosis and diminished ovarian reserve and in men, impaired spermatogenesis, obstructive azoospermia and varicocele. In a substantial proportion of couples, no cause is identified in either partner, and increasing attention has turned to environmental determinants that shape reproductive competence in both sexes long before conception [2,3]. Among these, endocrine-disrupting chemicals (EDCs) and a broader family of emerging pollutants—micro- and nanoplastics, per- and polyfluoroalkyl substances (PFASs), brominated and organophosphate flame retardants, and pharmaceuticals and personal-care products (PPCPs)—have become a central concern of molecular toxicology [2,3,4,5,6].

EDCs are exogenous substances, or mixtures, that interfere with any aspect of hormone action—synthesis, secretion, transport, receptor binding, or metabolism [2,3]. The category overlaps substantially with what environmental chemists term emerging pollutants: synthetic or naturally occurring contaminants that are increasingly detected in environmental and biological matrices, are frequently unregulated, and whose toxicological profiles are still being defined [4,5]. The reproductively relevant members of this group include plasticiser phthalates; polycarbonate and epoxy-resin monomer bisphenol A (BPA) and its analogues bisphenol S (BPS) and F (BPF); persistent per- and polyfluoroalkyl substances (PFASs); organophosphate, pyrethroid and carbamate pesticides; legacy organochlorines polychlorinated biphenyls (PCBs) and dioxins; polybrominated diphenyl ether (PBDE) and organophosphate flame retardants; pharmaceutical and personal-care residues sustained in surface water by continuous wastewater input; and, most recently, micro- and nanoplastics, which are both pollutants in their own right and mobile carriers of adsorbed EDCs [4,6,7,8,9].

What unites these structurally diverse xenobiotics is not a single target but a limited repertoire of molecular lesions. They perturb hormone-receptor signalling at low, physiologically relevant doses, sometimes with non-monotonic dose–response relationships [10]; they interfere with gene expression along the hypothalamic–pituitary–gonadal axis; they inhibit steroidogenic enzymes; they generate reactive oxygen species (ROS) and mitochondrial stress; they remodel the epigenome; and they intersect with metabolic and immune pathways [2,3,10,11,12,13]. These same lesions underlie both the loss of gamete and embryo competence and, over longer latencies, the initiation and progression of hormone-dependent reproductive cancers [5,14,15].

Despite the extensive literature, four gaps limit the utility of existing syntheses. First, fertility impairment and reproductive carcinogenesis are reviewed separately, although the molecular lesions underlying them are largely shared and differ principally in latency. Second, mechanistic frameworks have been built around persistent, bioaccumulative compounds, so pollutants that achieve chronic exposure through continuous environmental replenishment rather than through persistence—micro-/nanoplastics and PPCPs—sit awkwardly outside them. Third, mechanistic reviews rarely grade the certainty of the human evidence they cite, leaving readers unable to distinguish associations supported by multiple concordant cohorts from those resting on a single small study. Fourth, both the experimental and the epidemiological studies remain dominated by single-compound, single-generation designs, an approach that recent multi-generational work shows to be inadequate: descendant phenotypes may involve compensatory molecular responses qualitatively different from those of the exposed generation, and are therefore missed by endpoints chosen from F1 biology [16].

This review aims to establish the molecular toxicology that connects exposure to emerging pollutants with both impaired human fertility and hormone-dependent reproductive carcinogenesis and to state how securely each link is supported by human data. We synthesise the mechanisms through which structurally unrelated xenobiotics converge on shared molecular targets; we trace those targets forward into the cellular damage that impairs gametes and the early embryo and, over longer latencies, into malignancy; and we grade the certainty of the human evidence for each compound class, including the pseudo-persistent pollutants that conventional persistence-based frameworks accommodate poorly. Section 2, Section 3, Section 4,Section 5, Section 6 and Section 7 follow this sequence from exposure through mechanism to outcome and mitigation.

## 2. Occurrence, Sources and Human Exposure to Reproductively Active Emerging Pollutants

### 2.1. Chemical Classes, Environmental Sources and Rationale for Inclusion

Reproductively relevant emerging pollutants span several physicochemical families with distinct sources and environmental behaviours. The classes reviewed here were selected on three criteria: a high production volume with frequent detection in human biomonitoring programmes; documented reproductive or endocrine activity at concentrations within the range of human exposure; and mechanistic relevance to the pathways developed in Section 3. Classes meeting these criteria but lacking human reproductive data are retained and identified as such, since their omission would misrepresent the evidence landscape.

Phthalates, di-esters of phthalic acid such as di-(2-ethylhexyl) phthalate (DEHP), are non-covalently bound plasticisers that leach from PVC, food packaging, medical devices and personal-care products; being non-persistent, they are metabolised rapidly to monoester metabolites (e.g., MEHP, MnBP, MEOHP) [17,18]. Their inclusion is justified by near-universal urinary detection and by consistent anti-androgenic activity. Bisphenols (BPA, BPS, BPF) are polymer building blocks in polycarbonate plastics, epoxy-resin can linings and thermal paper [19,20]; they merit attention for both their intrinsic activity and because their substitutes retain it. PFASs, by contrast, are exceptionally persistent, water- and lipid-repellent surfactants that resist metabolism and bioaccumulate over years, contaminating drinking water, food packaging and firefighting foams [21,22]; their decade-long half-lives make internal dose a cumulative record of lifetime exposure. Pesticides—organophosphates, pyrethroids and carbamates—enter the population through diet and occupational agricultural exposure [23] and provide the clearest occupational evidence of male reproductive harm. Legacy PCBs and dioxins, though banned decades ago, persist in sediments and the food chain and bioaccumulate in adipose tissue [23,24,25], as do PBDE and organophosphate flame retardants used in textiles, foams and electronics [26]; these classes are informative because their declining exposure trends permit inference that recent, non-persistent exposures cannot.

Pharmaceuticals and personal-care products (PPCPs) constitute a chemically heterogeneous class that enters surface waters continuously through wastewater treatment plant effluent, since conventional treatment removes many of these compounds only partially; systematic surveys document the widespread occurrence of analgesics, antimicrobials and antibiotics across a decade of monitoring [6]. They warrant inclusion for a reason distinct from the classes above: their environmental half-lives are often short, but continuous replenishment sustains chronic low-concentration exposure, a pseudo-persistence that achieves the same exposure profile as bioaccumulation by a different route. Because many were designed to be biologically active at low doses in humans, their potential to perturb endocrine signalling at environmentally relevant concentrations is a priori plausible.

Micro- and nanoplastics (MNPs) have emerged as a distinct and rapidly growing class of pollutants. Formed by the mechanical, photo-oxidative and biological fragmentation of bulk plastics, MNPs are now documented in tap water, indoor dust, seafood and, critically, in human placenta, blood and other tissues [7,8,9,27,28]. Beyond their intrinsic particle toxicity, MNPs adsorb and concentrate hydrophobic co-contaminants—including bisphenols, phthalate esters and persistent organics—acting as vectors that can amplify combined reproductive toxicity [8,9].

### 2.2. Routes of Human Exposure

The route of exposure determines internal dose and, for particulate pollutants, the tissues initially encountered. It differs systematically between classes. Dietary ingestion dominates for phthalates, bisphenols and legacy organochlorines, with migration from food-contact materials and bioaccumulation through the food chain as the principal pathways [29,30]. Drinking water is the leading route for PFASs in contaminated catchments and, together with dietary residues, for PPCPs [6,21]. The inhalation of indoor air and ingestion of settled house dust are major and frequently underestimated routes for flame retardants, semi-volatile plasticisers and micro- and nanoplastics, all of which partition into the indoor compartment [27,31,32]. For particles, inhalation additionally permits access to systemic circulation across the alveolar barrier, bypassing first-pass hepatic metabolism [32,33]. Dermal absorption is significant for personal-care-product constituents, including triclosan and certain phthalates, and for thermal-paper handling as a source of bisphenols [29,30].

**Figure 1:**
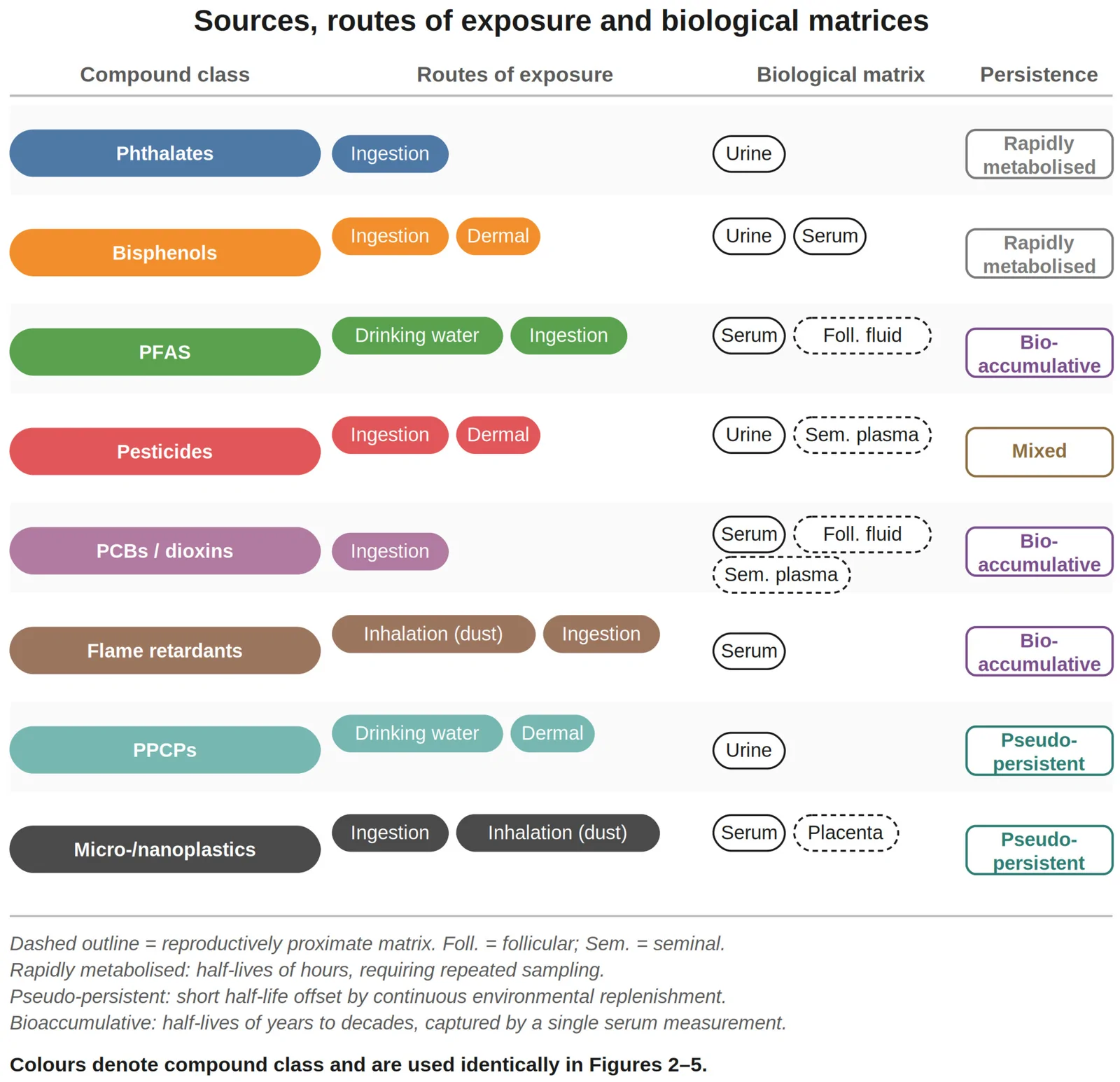
Sources, routes of exposure and biological matrices for the reproductively active emerging pollutants reviewed here. Colours denote compound class and are used consistently in Figure 2, Figure 3, Figure 4 and Figure 5; the persistence axis distinguishes bioaccumulative compounds (PFASs, PCBs/dioxins, PBDEs) from pseudo-persistent pollutants sustained by continuous environmental replenishment (PPCPs, micro-/nanoplastics) and from rapidly metabolised compounds (phthalates, bisphenols). Route of exposure is indicated by the labelled route pills in the second column; matrices are grouped by whether they are systemic or reproductively proximate.

These distinctions matter for interpretation. The route governs the metabolic processing a compound undergoes before it reaches reproductive tissue. Exposure biomarkers measured in urine or serum may therefore under- or over-represent the dose delivered to the gonad, depending on the route by which exposure occurred [30,34]. For MNPs in particular, particle size and route together determine biodistribution, and the ability of nanoscale particles to cross biological barriers underlies the size-dependent toxicity described in Section 5.1 [32,33]. Figure 1 summarises the classes, sources, routes and matrices in which each class is measured.

**Figure 2:**
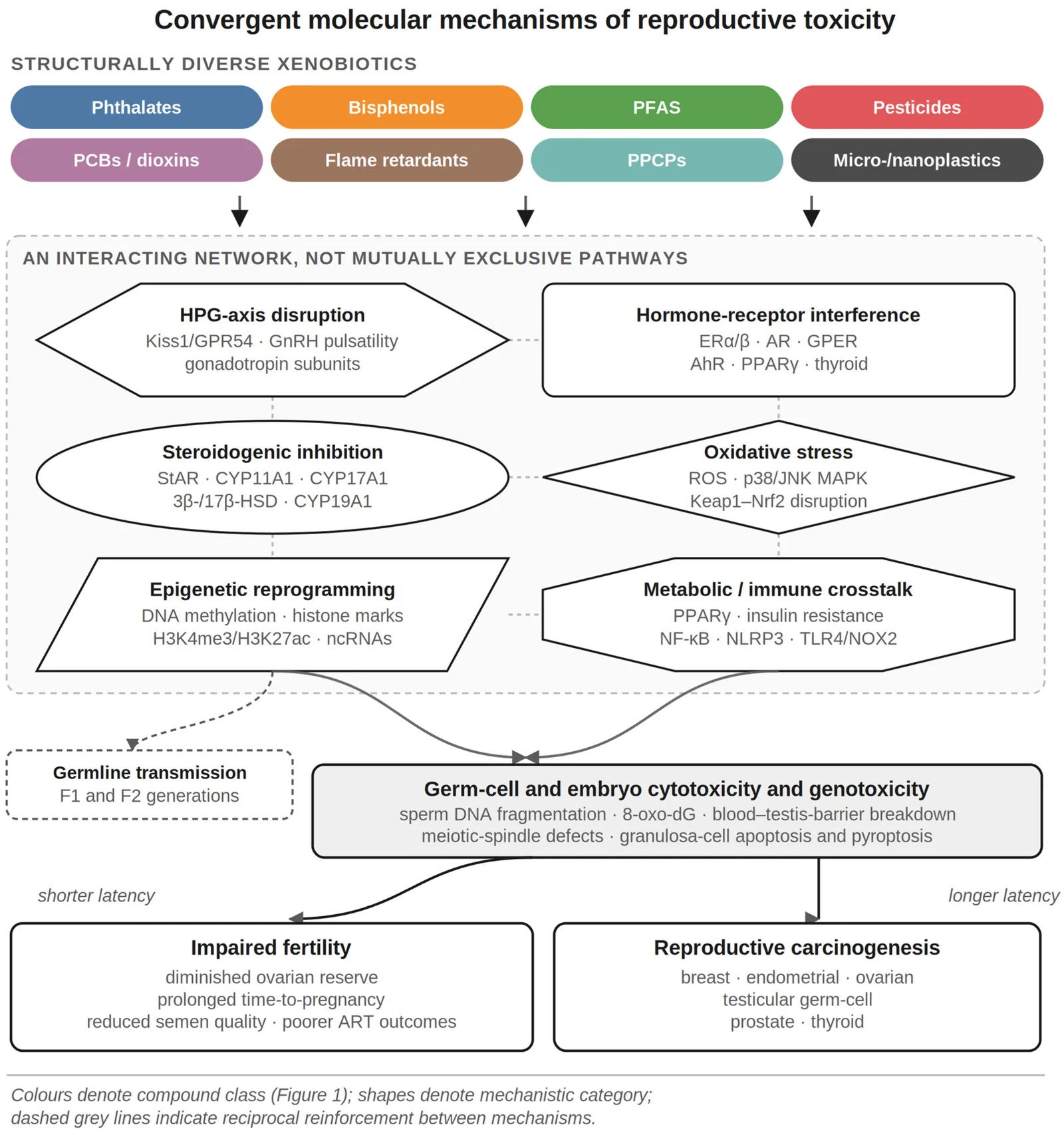
Convergent molecular mechanisms of emerging-pollutant reproductive toxicity. Colours denote compound class as defined in Figure 1; shapes denote mechanistic category; dashed grey lines indicate reciprocal reinforcement between mechanisms. Structurally diverse xenobiotics act through a shared network—HPG-axis disruption (kisspeptin/GPR54, GnRH pulsatility, gonadotropin subunit expression), hormone-receptor interference (ER/AR/GPER/AhR/PPARγ), steroidogenic-enzyme inhibition (StAR, CYP19A1, 3β-/17β-HSD), ROS generation with Keap1–Nrf2 disruption and mitochondrial dysfunction, epigenetic reprogramming (DNA methylation, histone marks, ncRNAs) and metabolic/immune crosstalk (NF-κB, NLRP3)—that converge on germ-cell and embryo cytotoxicity and genotoxicity and, over longer latencies, reproductive carcinogenesis. Germ-line epigenetic changes may be transmitted across generations.

**Figure 3:**
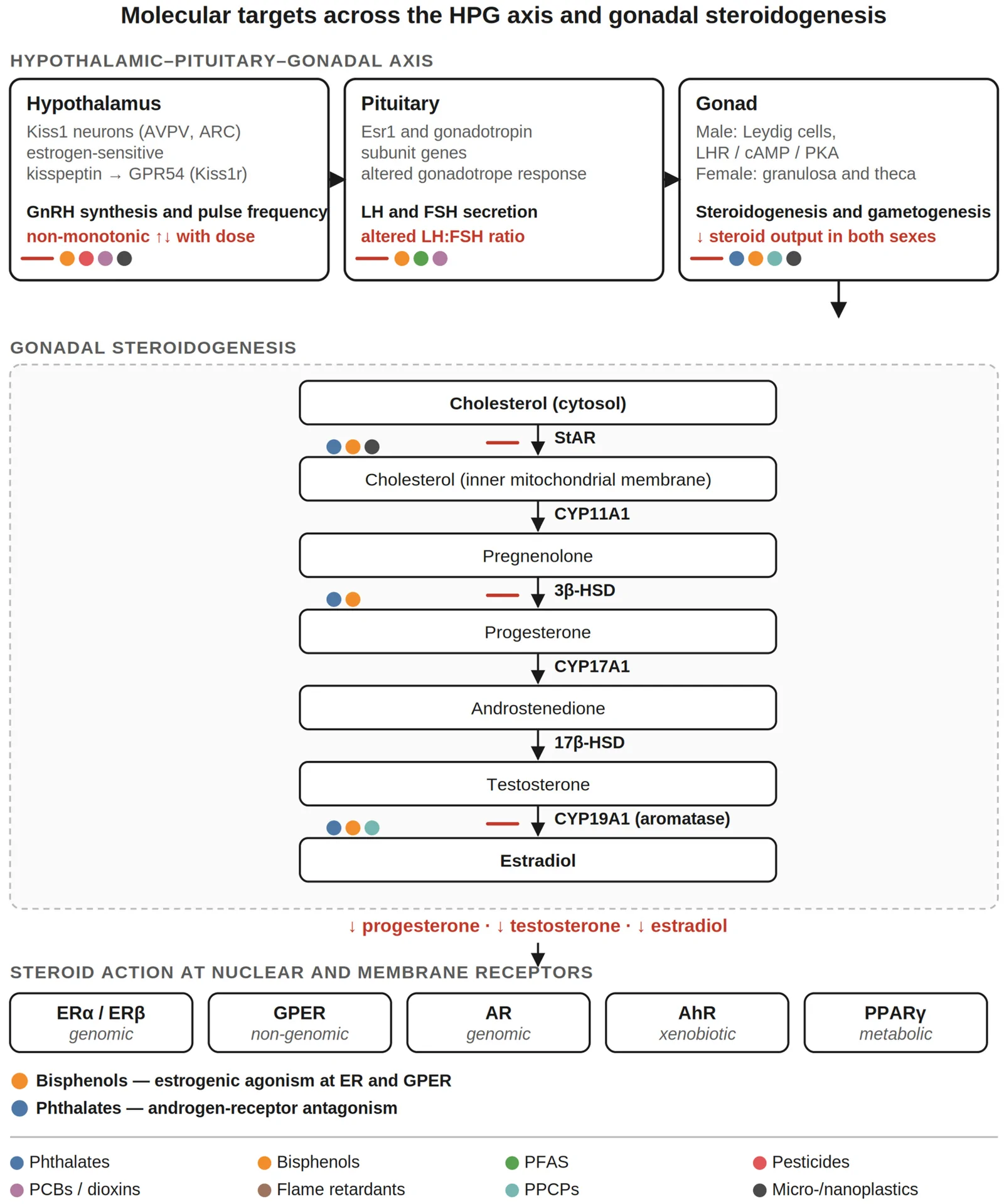
Molecular targets of emerging pollutants across the hypothalamic–pituitary–gonadal axis and gonadal steroidogenesis. Colours denote compound class as defined in Figure 1; red bars indicate inhibition and red text marks the resulting change in hormone output. Pollutants perturb kisspeptin/GPR54 control of GnRH pulsatility and pituitary gonadotropin subunit expression, suppress luteinizing-hormone-driven, StAR-mediated cholesterol transport, and inhibit the CYP11A1–CYP17A1–3β-/17β-HSD–CYP19A1 (aromatase) enzyme cascade, lowering progesterone, testosterone and estradiol. The resulting steroids act on nuclear and membrane receptors (ERα/β, GPER, AR, AhR, PPARγ), at which bisphenols exert estrogenic agonism and phthalates exert androgen-receptor antagonism.

**Figure 4:**
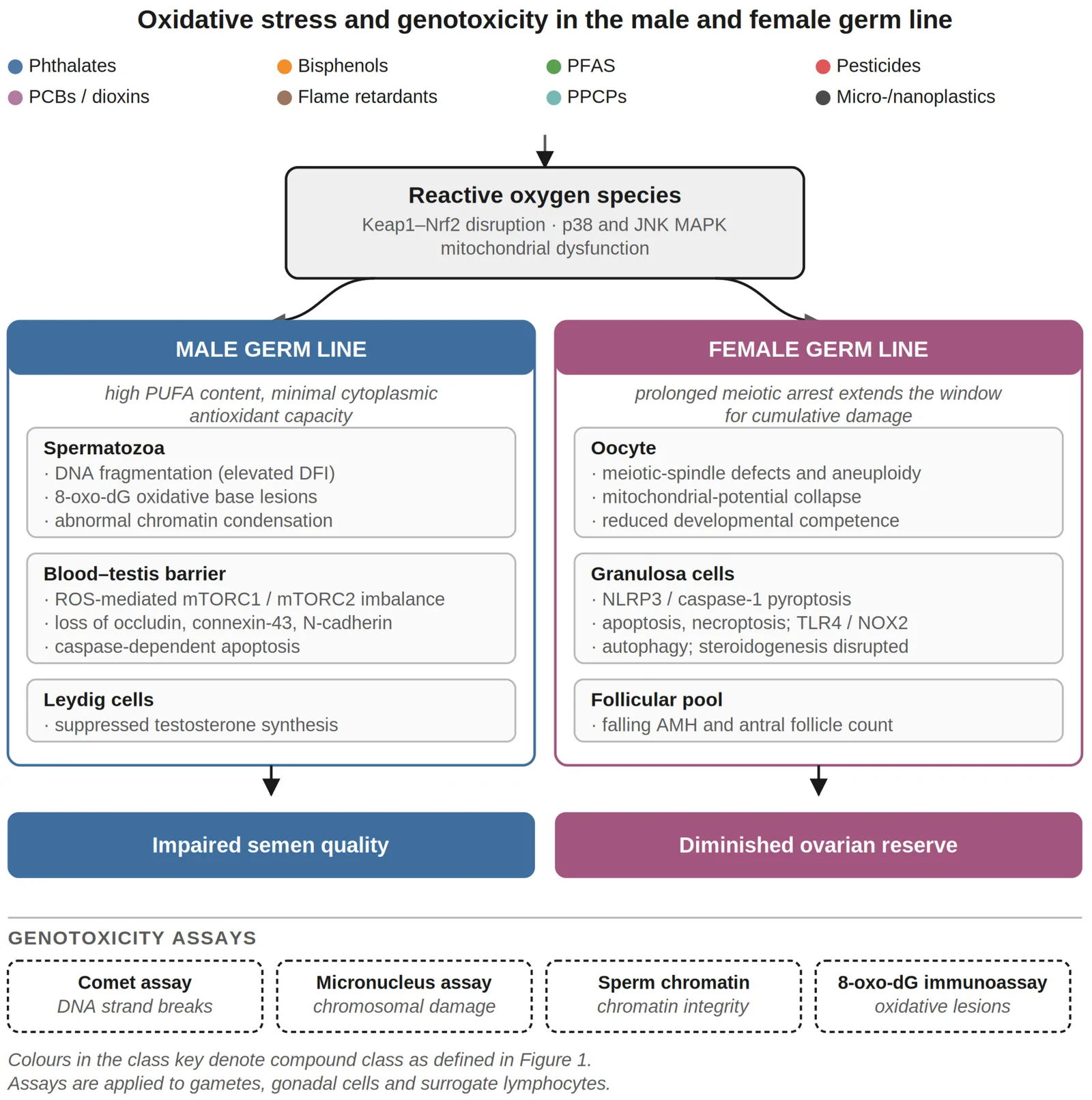
Oxidative stress and genotoxicity in the male and female germ line. Colours denote compound class as defined in Figure 1. Pollutant-driven ROS generation with Keap1–Nrf2 disruption and p38/JNK MAPK activation damages the blood–testis barrier, spermatozoa (DNA fragmentation, 8-oxo-dG, apoptosis) and Leydig cells in the male and granulosa cells (NLRP3/caspase-1 pyroptosis, apoptosis, autophagy, necroptosis) and oocytes (meiotic-spindle and mitochondrial defects) in the female, yielding impaired semen quality and diminished ovarian reserve. Blood–testis-barrier disruption proceeds through ROS-mediated imbalance of the mTORC1 and mTORC2 complexes as described in Section 4.1. Genotoxic damage is quantified by the comet, micronucleus, sperm-chromatin-structure and 8-oxo-dG assays.

**Figure 5:**
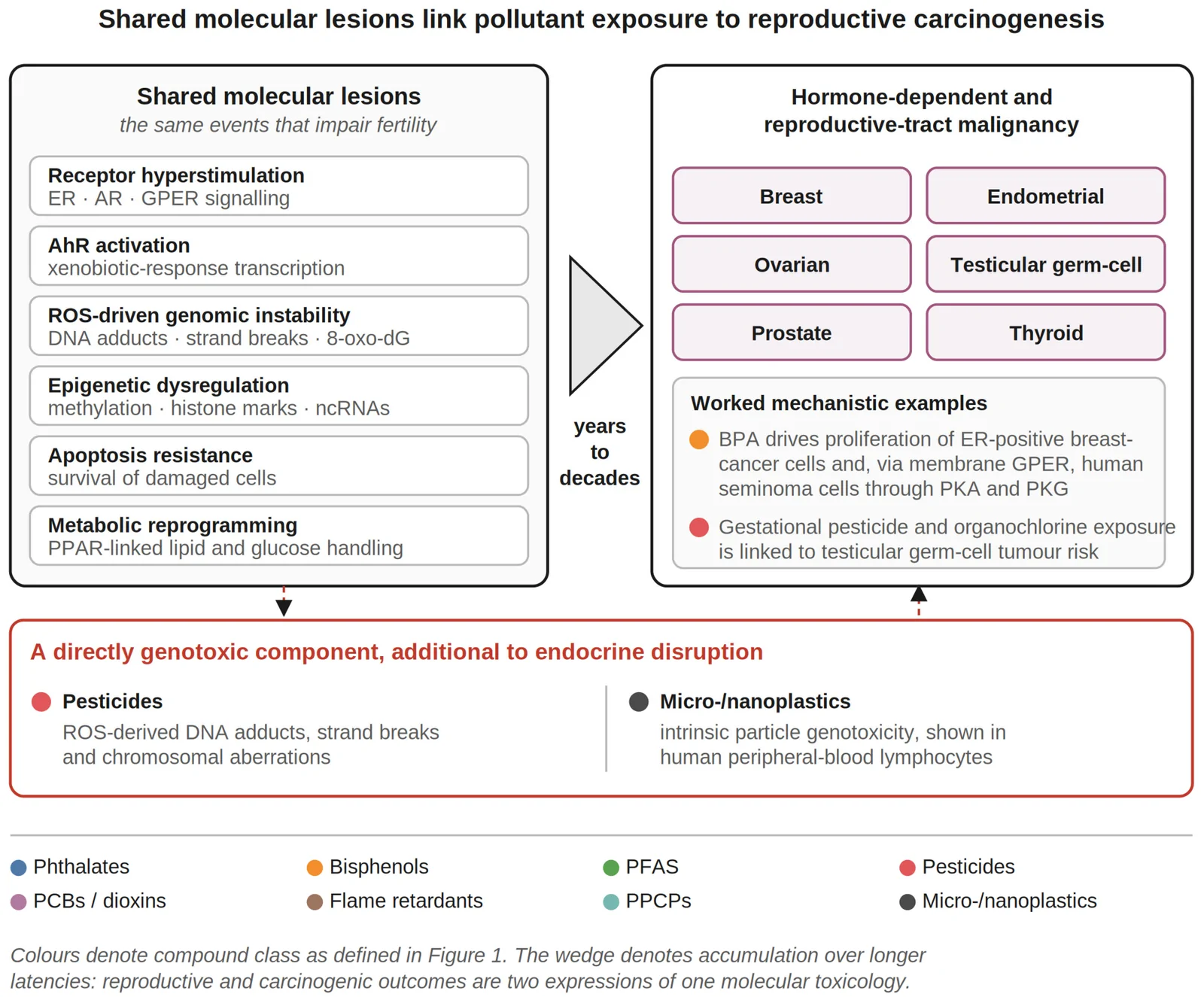
Shared molecular lesions link emerging-pollutant exposure to reproductive and hormone-dependent carcinogenesis. Colours denote compound class as defined in Figure 1. Receptor hyperstimulation (ER/AR/GPER), AhR activation, ROS-driven genomic instability, epigenetic dysregulation, apoptosis resistance and metabolic reprogramming accumulate over longer latencies to promote breast, endometrial, ovarian, testicular germ-cell, prostate and thyroid cancers, each of which is discussed in Section 5.3; pesticides and micro-/nanoplastics add a directly genotoxic component, shown in the red-outlined panel, whose red dashed arrows denote its contribution to both the shared lesions and the malignant endpoints.

### 2.3. Internal Dose, Biological Matrices and Analytical Identification

Because most of these pollutants are ingested, inhaled or dermally absorbed at low chronic doses, exposure assessment relies on the measurement of internal dose in biological matrices. Human biomonitoring programmes—exemplified by the U.S. National Health and Nutrition Examination Survey (NHANES)—have used isotope-dilution liquid chromatography–tandem mass spectrometry (LC–MS/MS) to quantify urinary phthalate metabolites and free and conjugated BPA at population scale, establishing near-ubiquitous detection [17,19]. Matrix selection follows compound behaviour: urine is appropriate for rapidly metabolised, water-soluble compounds and their conjugates, including phthalate metabolites, bisphenols, triclosan and antibiotic residues; serum is required for lipophilic, protein-bound and persistent compounds including PFASs, PCBs, dioxins and PBDEs [21,35,36,37].

Reproductively proximate matrices are increasingly analysed—phthalate and bisphenol metabolites in urine and serum, PFASs in serum and follicular fluid, and organochlorines in seminal plasma and follicular fluid—allowing exposure to be related directly to gamete and embryo endpoints [18,21,35,38]. These matrices are of particular value because they remove one inferential step between measured exposure and the tissue in which the mechanisms of Section 3 operate.

Analytical identification differs by pollutant class. Non-persistent phthalates and bisphenols, with urinary half-lives of hours, require careful timing and, ideally, repeated sampling to avoid exposure misclassification; PFASs and organochlorines, with half-lives of years, are more reliably captured by single serum measurements [21,35]. PPCPs share the short-half-life problem of non-persistent compounds, so single-spot measurements characterise continuous exposure poorly [36]. For particulate pollutants, identification depends on vibrational-spectroscopy and thermal-degradation methods—μ-Fourier-transform infrared and Raman spectroscopy, and pyrolysis–gas chromatography–mass spectrometry—which resolve polymer type, particle size and abundance in water, dust and tissue [8,27,28]. These occurrence and identification data define the exposure term that the molecular mechanisms in the following section act upon.

## 3. Molecular Mechanisms and Class-Specific Evidence

Endocrine-disrupting emerging pollutants impair reproduction through a limited set of convergent molecular mechanisms that operate across the hypothalamic–pituitary–gonadal (HPG) axis, the gonads and the early embryo. These are best understood not as mutually exclusive pathways but as an interacting network in which axis-level disruption, receptor interference, enzyme inhibition, oxidative stress, epigenetic change and metabolic–immune crosstalk reinforce one another [2,3,11]. This section sets out each mechanism in turn, distinguishing male and female effects within each, and then presents the class-specific human evidence in Section 3.7. Table 1 provides a class-wise entry point for readers who prefer to approach the material by compound rather than by mechanism, and Figure 2 summarises the convergence.

### 3.1. Disruption of Hypothalamic–Pituitary–Gonadal Signalling

Reproductive function depends on pulsatile gonadotropin-releasing hormone (GnRH) secretion from hypothalamic neurons, which drives the pituitary release of LH and FSH and thereby gonadal steroidogenesis and gametogenesis. The axis is held in balance by steroid feedback acting through ER and AR expressed in the hypothalamus and pituitary, an architecture that makes it intrinsically vulnerable to compounds that mimic or block steroid signalling [3,13].

The kisspeptin system is a principal point of vulnerability. Kisspeptin, encoded by Kiss1, signals through GPR54 (Kiss1r) to control GnRH synthesis and pulse frequency, and Kiss1 neurons of the anteroventral periventricular and arcuate nuclei are themselves estrogen-sensitive. Xenoestrogens acting on these neurons therefore alter the drive to GnRH neurons rather than acting on GnRH neurons directly. Experimental exposures produce dose-dependent and frequently non-monotonic changes in Kiss1/GPR54 and GnRH expression, with low and high doses of the same compound reported to act in opposite directions [13]. Such non-monotonicity is mechanistically expected where a compound engages several receptor systems with differing affinities, and is one reason why high-dose testing predicts low-dose outcomes poorly [10].

At the pituitary, developmental exposure alters the expression of Esr1 and of gonadotropin subunit genes, changing the responsiveness of gonadotropes to a given GnRH pulse pattern and hence the LH:FSH ratio reaching the gonad [13]. Because the timing and amplitude of gonadotropin exposure govern follicular recruitment in the ovary and Sertoli-cell function in the testis, disturbances at this level propagate to gonadal outcomes even when gonadal tissue is not itself a direct target—a point that matters for interpretation, since gonadal phenotypes are frequently attributed to direct gonadal action without the axis having been examined.

In the male germ line, reduced or disordered LH pulsatility limits Leydig-cell testosterone output, and because spermatogenesis requires high intratesticular testosterone, modest reductions translate into disproportionate deficits in germ-cell maturation; altered FSH signalling additionally compromises Sertoli-cell support of the seminiferous epithelium. In the female germ line, altered LH:FSH ratios disturb follicular recruitment and dominance selection, and disrupted feedback can abolish the preovulatory LH surge, producing anovulatory cycles independently of any direct ovarian toxicity. These axis-level effects act in concert with the direct gonadal mechanisms described in Section 3.2 and Section 3.3.

### 3.2. Nuclear and Membrane Hormone-Receptor Interference

A second and better-characterised mechanism is direct interaction with hormone receptors. Bisphenols and several pesticides bind to the nuclear estrogen receptors ERα (ESR1) and ERβ (ESR2), acting as agonists or antagonists to distort estrogen-dependent transcription that governs folliculogenesis and endometrial receptivity [2,11,39]. Phthalate monoesters, by contrast, are predominantly anti-androgenic, antagonising the androgen receptor (AR) and suppressing the Leydig-cell testosterone programme and Sertoli-cell support of spermatogenesis [11,18].

The functional consequence of receptor binding depends on the ligand and the cellular context rather than on affinity alone. A compound may act as an agonist, recruiting coactivators to estrogen- or androgen-response elements and initiating transcription; as an antagonist, occupying the ligand-binding domain while favouring corepressor recruitment; or as a selective modulator whose activity differs between tissues according to the local coregulator complement. Because most xenoestrogens bind ER with affinity orders of magnitudes below that of 17β-estradiol, their significance is derived not from potency but from chronic exposure, mixture effects, and action during developmental windows when endogenous hormone concentrations are low and the receptor system is being organised. Anti-androgenic compounds may additionally act indirectly, inhibiting steroidogenic enzymes or accelerating androgen clearance, so that AR-dependent transcription falls without direct receptor occupancy [3,13].

Many of these compounds also signal through the membrane-associated G-protein-coupled estrogen receptor (GPER), triggering rapid, non-genomic PKA/PKG, ERK1/2 and PI3K–Akt cascades at picomolar-to-nanomolar concentrations—a route that explains low-dose and non-monotonic responses that classical nuclear-receptor models cannot [10,39,40]. PCBs, dioxins and PFASs additionally perturb thyroid-hormone receptor signalling and the aryl-hydrocarbon receptor (AhR), while PFASs and phthalates engage peroxisome-proliferator-activated receptor γ (PPARγ), linking endocrine disruption to lipid and metabolic programming [3,15]. This multi-receptor promiscuity means that a single xenobiotic can simultaneously modulate several transcriptional programmes central to gametogenesis and implantation.

In the male germ line, the dominant receptor-mediated lesion is AR antagonism with consequent suppression of the Leydig-cell androgenic programme and impaired Sertoli-cell support; in the female germ line, it is inappropriate ER and GPER activation in granulosa cells and endometrium, distorting folliculogenesis and receptivity. The same compound may therefore produce mechanistically distinct injuries in the two sexes.

### 3.3. Disruption of Steroidogenesis

Beyond receptor mimicry, emerging pollutants directly inhibit the steroidogenic machinery. Steroid synthesis begins with the transport of cholesterol into mitochondria by the steroidogenic acute regulatory (StAR) protein, followed by the sequential action of the side-chain cleavage enzyme CYP11A1 (P450scc), CYP17A1 (P450c17), 3β-hydroxysteroid dehydrogenase (3β-HSD), 17β-HSD and the aromatase CYP19A1, which converts androgens to estrogens [41]. Phthalate metabolites and bisphenols down-regulate StAR expression and inhibit CYP19A1/aromatase and 3β-HSD, lowering the synthesis of progesterone, estradiol and testosterone [11,18,41]. In experimental systems, chronic exposure to polystyrene microplastics likewise suppresses StAR and steroidogenic enzymes through inhibition of the luteinizing-hormone–receptor (LHR)/cAMP/PKA axis in Leydig cells, reducing circulating testosterone, LH and FSH [8,42].

Suppression of StAR restricts cholesterol delivery to the inner mitochondrial membrane and therefore limits steroid output at its first committed step, whereas altered aromatase expression shifts the local androgen-to-estrogen balance without necessarily changing total steroid flux; the two lesions are not interchangeable. In the male germ line, the consequence is impaired Leydig-cell testosterone synthesis with secondary disturbance of Sertoli-cell function and blood–testis-barrier integrity. In the female germ line, granulosa and theca cells are the corresponding targets, and disruption of granulosa-cell steroidogenesis compromises folliculogenesis and oocyte quality (Figure 3).

### 3.4. Oxidative Stress and Mitochondrial Dysfunction

A further convergent mechanism is the generation of reactive oxygen species (ROS) and consequent mitochondrial dysfunction. Phthalates, bisphenols, pesticides and micro-/nanoplastics all increase ROS production while depleting antioxidant defences, tipping the redox balance toward oxidative damage of lipids, proteins and DNA [8,12,43,44,45]. Mechanistically, ROS activate stress-responsive MAPK cascades—particularly p38 and JNK—and overwhelm the cytoprotective Kelch-like ECH-associated protein 1 (Keap1)–the nuclear-factor-erythroid-2-related-factor-2 (Nrf2) axis that normally induces antioxidant and phase-II detoxification genes [44,46,47].

In the male germ line, this ROS surge drives lipid peroxidation of the sperm membrane, sperm DNA fragmentation and disruption of the blood–testis barrier; spermatozoa are particularly vulnerable because their plasma membranes are rich in polyunsaturated fatty acids and their cytoplasmic antioxidant capacity is minimal. In the female germ line, ROS provoke granulosa-cell apoptosis, mitochondrial-membrane-potential collapse and meiotic errors in oocytes, with the long meiotic arrest of the human oocyte providing an extended window for cumulative oxidative damage [43,48,49]. BPA in particular has been proposed to act through oxidative stress as a unifying leitmotif of its pleiotropic reproductive toxicity, coupling mitochondrial dysfunction to apoptosis in both sexes [12].

### 3.5. Epigenetic Reprogramming and Transgenerational Inheritance

Emerging pollutants also act at the epigenetic level, altering gene expression without changing DNA sequence. Documented modifications include changes in DNA methylation, covalent histone marks (for example, exposure-induced H3K4 trimethylation at hormone-responsive loci) and altered expression of non-coding RNAs, including microRNAs [3,15].

Recent single-cell work has identified a further route to the epigenome that does not depend on receptor binding. In pubertal mice exposed to polystyrene micro- and nanoplastics, granulosa cells were the principal ovarian target of the 100 nm fraction; only nanoscale particles were internalised, producing cell-cycle arrest, necroptosis and steroidogenic dysfunction [50]. Mechanistically, the particles remodelled the F-actin cytoskeleton and increased cell stiffness, and this mechanical perturbation was accompanied by altered H3K4me3 and H3K27ac deposition and changed chromatin accessibility, with integrated ATAC-seq and RNA-seq analyses implicating STAT1 as a key transcriptional regulator [50]. Cytoskeletal mechanotransduction therefore provides a route by which a physically, rather than chemically, acting contaminant reaches the transcriptome, distinct from the receptor-mediated pathways that dominate the classical EDC literature and consistent with the granulosa-cell tropism observed in earlier rodent studies [43,49] and with the altered cytoskeletal protein expression reported after microplastic exposure in rat ovary [51].

Because the germ line is epigenetically reprogrammed during critical developmental windows, exposures during foetal, perinatal and peripubertal life can imprint durable changes on the gametes; experimental models demonstrate that some of these marks are transmitted across generations, extending reproductive consequences beyond the directly exposed individual [3,15].

The assumption that descendant phenotypes are attenuated versions of the exposed-generation phenotype requires qualification. Following perinatal polystyrene microplastic exposure, F1 males showed suppressed testosterone synthesis with reduced StAR and elevated GRP78, indicating steroidogenic impairment coupled to endoplasmic-reticulum stress; F2 males showed reduced sperm counts and increased morphological abnormalities, but with upregulated StAR and SRSF1, suggesting that the compensatory modulation of steroidogenesis and RNA splicing partially offsets the ancestral insult [16]. The F2 molecular signature is therefore qualitatively different from that of F1, not merely weaker—a finding with direct consequences for multi-generational risk assessment, since endpoints selected from exposed-generation biology may fail to detect the descendant phenotype. This epigenetic memory provides a molecular explanation for the delayed and heritable reproductive effects observed after developmental EDC exposure and is increasingly implicated in the early exposure–long-term memory model of pollutant-associated carcinogenesis [15].

### 3.6. Crosstalk with Metabolic and Immune Signalling

Finally, these pollutants intersect with broader metabolic and immune networks that are themselves determinants of fertility. Through PPARγ, ER and AhR signalling, they promote adipogenesis, insulin resistance and dyslipidaemia, contributing to metabolic phenotypes such as obesity and polycystic ovary syndrome that independently impair reproduction [3,15]. In parallel, particulate and chemical pollutants activate innate-immune and inflammatory pathways—NF-κB signalling, the NLRP3 inflammasome and TLR4/NADPH-oxidase-2 (NOX2) cascades—that disturb the immune tolerance required for implantation and early pregnancy maintenance and that drive the pyroptotic remodelling of reproductive tissues [43,49]. The convergence of axis-level, endocrine, oxidative, epigenetic, metabolic and immune perturbations forms the coherent molecular framework linking exposure to impaired fertility summarised in Figure 2.

### 3.7. Class-Specific Mechanistic and Human Evidence

The following synthesis integrates, for each major pollutant class, the dominant molecular mechanism with the human reproductive evidence and its certainty. Effect estimates from the underlying epidemiology are consistent in direction across regions and study designs. The certainty ratings below are assigned by the authors following GRADE domains—risk of bias, inconsistency, indirectness, imprecision and publication bias—applied to the body of evidence for each outcome; they are our assessments rather than ratings reported in the primary studies.

#### 3.7.1. Phthalates

Phthalates act principally as anti-androgens and steroidogenic inhibitors, suppressing StAR and aromatase and elevating oxidative stress in gametes [11,18,41]. In women, higher urinary DEHP-metabolite concentrations are inversely associated with anti-Müllerian hormone and antral follicle count and with prolonged time-to-pregnancy, and predict reduced oocyte yield, poorer embryo quality and lower implantation and live-birth rates in assisted reproductive technology (ART) [52,53]. In men, phthalate exposure is consistently associated with reduced sperm concentration and motility, increased sperm DNA fragmentation and aneuploidy, and lower circulating testosterone [18]. A systematic review of 38 studies published between 2014 and 2024 reaches the same conclusion for sperm concentration, motility and morphology [54] (Table 2).

#### 3.7.2. Bisphenols (BPA, BPS, BPF)

Bisphenols combine ER/GPER-mediated estrogenicity with anti-androgenic activity, aromatase inhibition and ROS-driven mitochondrial dysfunction [11,12,39]. Higher BPA exposure is associated with reduced ovarian reserve and, in ART, with fewer mature oocytes, lower fertilisation rates and reduced implantation and live-birth rates [55,56,57]. In men, BPA is linked to impaired sperm motility and morphology and to sperm DNA damage, with less consistent effects on concentration [48,58,59,60]. Critically, the substitutes BPS and BPF display hormonal and oxidative activity comparable to BPA, such that regrettable substitution preserves rather than removes the molecular hazard [20] (Table 3).

#### 3.7.3. Per- and Polyfluoroalkyl Substances (PFASs)

PFAS are persistent, bioaccumulative disruptors that engage PPARγ and ESR1, induce mitochondrial dysfunction and alter thyroid signalling [3,15]. Higher circulating perfluorooctane sulfonate (PFOS) and perfluorooctanoic acid (PFOA) are associated with reduced fecundability and longer time-to-pregnancy, and, in ART, with lower oocyte yield, poorer embryo quality and reduced implantation and live-birth rates [21,38]. Male exposure is associated with reduced sperm motility, abnormal morphology and altered testosterone [22]. Meta-analysis of human studies links PFOA and PFNA to lower circulating testosterone [61], and measurement of PFAS directly in seminal plasma, rather than in plasma alone, strengthens the association with reduced progressive motility [62]. Blood–testis-barrier disruption, oxidative stress and altered sperm DNA methylation are the mechanisms most often proposed [63] (Table 4).

#### 3.7.4. Pesticides

Organophosphate, pyrethroid and carbamate pesticides act through combined endocrine disruption, oxidative DNA damage and epigenetic change, and are among the pollutants most clearly linked to both reproductive impairment and carcinogenesis [15]. Occupational and environmental exposure in women is associated with longer time-to-pregnancy, higher miscarriage risk and reduced ovarian-reserve markers, and with lower implantation rates in ART; in men—especially agricultural workers—exposure is robustly associated with impaired sperm concentration, motility and morphology [15]. Mechanistically, pesticides generate ROS that produce oxidative DNA lesions (8-oxo-dG), DNA adducts and strand breaks, and perturb DNA-methylation and microRNA profiles—changes that bridge reproductive toxicity and the elevated risk of hormone-dependent and germ-cell tumours discussed in Section 5 [15] (Table 5).

#### 3.7.5. Polychlorinated Biphenyls (PCBs) and Dioxins

Evidence for PCBs and dioxins derives largely from legacy cohorts and accidental high-exposure events. These lipophilic organochlorines act predominantly through AhR activation and thyroid disruption and bioaccumulate over decades [3,23]. Exposure is associated with delayed time-to-pregnancy, menstrual irregularity and reduced semen quality, with limited ART data suggesting lower implantation and live-birth rates [23,24,25]. Prenatal organochlorine exposure (for example, DDT/DDE) has intergenerational effects, reducing fecundity in exposed daughters [23] (Table 6).

#### 3.7.6. Flame Retardants (PBDEs and OPFRs)

Data on brominated (PBDE) and organophosphate flame retardants remain more limited. These compounds interfere with thyroid-hormone signalling critical to oocyte maturation and early development [26]. Higher PBDE exposure in women is associated with longer time-to-pregnancy and reduced ART success, while sparse male data suggest reduced sperm motility and abnormal morphology [26] (Table 7).

#### 3.7.7. Pharmaceuticals and Personal-Care Products (PPCPs)

PPCPs differ from the classes above in the mechanism by which chronic exposure is sustained: their environmental half-lives are often short, but continuous wastewater input maintains low-concentration exposure indefinitely [6]. The strength of the human evidence varies markedly between compounds within the class, and we distinguish them accordingly (Table 8).

Triclosan, an antimicrobial with both estrogenic and androgenic activity in vitro, is the PPCP for which human reproductive data are most developed. Among 511 women attending an infertility clinic, urinary triclosan was inversely associated with antral follicle count, though not with AMH, FSH or estradiol [37]. In the MIREC cohort, higher urinary triclosan was associated with prolonged time-to-pregnancy [64]. In men, environmental exposure has been associated with poorer semen quality [65] and reduced fecundability [66], and a systematic review and meta-analysis have synthesised the semen-quality evidence [67]. Effect sizes are generally moderate and dose–response relationships inconsistent, and a systematic review found no overall association with clinical infertility while identifying semen quality and ovarian reserve as susceptible endpoints. The evidence therefore supports subclinical effects on reproductive parameters rather than a demonstrated effect on fertility outcomes.

Antibiotic residues have been examined more recently. In a biomonitoring study of 986 reproductive-aged men in which 42 urinary antibiotics were quantified, both individual compounds and antibiotic mixtures were inversely associated with semen parameters; quantile g-computation indicated negative associations of the mixture with sperm concentration and with total and progressive motility, and the inverse associations of erythromycin and doxycycline with sperm concentration were stronger in younger men [36]. In the LIFE cohort, urinary antimicrobial biomarkers showed bidirectional associations with semen quality parameters but no association with sperm DNA damage [68]. Because exposure in these populations reflects therapeutic use as well as environmental and dietary intake, attributing effects specifically to environmental residues remains difficult.

Non-steroidal anti-inflammatory drugs illustrate the limits of the current evidence. Diclofenac is among the most frequently detected pharmaceuticals in surface waters, but to our knowledge, no epidemiological study has linked environmentally relevant exposure to semen quality, ovarian reserve or time-to-pregnancy. The available evidence is experimental: chronic exposure of mice to environmentally relevant doses of NSAID mixtures containing diclofenac, combined with 17α-ethinylestradiol, altered reproductive organ maturation, estrous cyclicity and spermiogenesis in F1 animals, with defects persisting into F2 alongside transcriptomic changes in testicular and ovarian pathways [69]. Higher-dose rodent studies of diclofenac alone report reduced testicular and epididymal weights and dose-dependent decreases in sperm count and motility, at doses well above environmental relevance. Diclofenac is therefore best regarded as a compound of mechanistic concern for which human reproductive epidemiology constitutes a clear gap rather than an established risk.

Mixture exposure is a defining feature of this class: PPCPs occur in aquatic environments as complex mixtures, and the experimental evidence above is derived from mixture designs precisely because single-compound exposure poorly represents environmental reality. Epidemiological methods capable of handling correlated multi-compound exposures, such as those applied in [36], are likely to be necessary for further progress.

## 4. Cytotoxicity and Genotoxicity in Germ Cells and the Early Embryo

The molecular lesions described above ultimately manifest as measurable cytotoxic and genotoxic damage to gametes, gonadal somatic cells and the pre-implantation embryo—the cellular substrate of impaired fertility (Figure 4).

### 4.1. Male Germ Line: DNA Fragmentation, Oxidative Lesions and Barrier Failure

In the male germ line, pollutant-induced ROS are the proximal cause of genotoxic damage. Elevated ROS produce sperm DNA fragmentation, measurable as an increased DNA fragmentation index, and oxidative base lesions such as 8-oxo-7,8-dihydro-2′-deoxyguanosine (8-oxo-dG), together with abnormal chromatin condensation and sperm aneuploidy [18,48]. Human biomonitoring links urinary BPA and phthalate metabolites to precisely these endpoints—reduced sperm concentration and motility, abnormal morphology, increased DNA fragmentation and altered chromatin structure [18,48,59,60]. At the tissue level, experimental micro-/nanoplastic exposure disrupts the blood–testis barrier through ROS-driven MAPK–Nrf2 signalling and an imbalance of the mTORC1/mTORC2 complexes, degrading tight-junction and gap-junction proteins (occludin, connexin-43, N-cadherin) and triggering caspase-dependent apoptosis of spermatogenic cells [44,46,47,70]. Genotoxicity is not confined to germ cells: polyethylene microplastics induce dose-dependent DNA damage and cytotoxicity in human peripheral-blood lymphocytes, confirming intrinsic particle genotoxic potential [71]. Human observational data are beginning to converge with these experimental findings: microplastics have been identified directly in human semen by Raman microspectroscopy [72], and, in a multi-site Chinese study, the number of polymer types detected in semen was inversely related to total sperm count, concentration and progressive motility [73].

### 4.2. Female Germ Line and Gonadal Soma: Meiotic Errors, Apoptosis and Pyroptosis

In females, comparable oxidative and inflammatory mechanisms damage the oocyte and its supporting granulosa cells. ROS accumulation provokes meiotic-spindle defects and aneuploidy in oocytes and collapses mitochondrial membrane potential, compromising developmental competence [12,43]. Granulosa cells are particularly vulnerable: microplastic and bisphenol exposure activates the NLRP3/caspase-1 inflammasome and TLR4/NOX2 axis and inhibits PI3K/AKT signalling, causing granulosa-cell apoptosis, pyroptosis and autophagy with disrupted steroidogenesis [43,49]. In juvenile animals, the same exposures raise the atretic follicle ratio and lower circulating estradiol and progesterone through oxidative and endoplasmic-reticulum stress [45], and impair ovarian function with altered cytoskeletal protein expression [51]—cellular correlates of the diminished ovarian reserve seen epidemiologically. Single-cell analysis has since confirmed granulosa cells as the principal ovarian target of nanoscale particles specifically, and identified cell-cycle arrest and necroptosis alongside the apoptotic and pyroptotic pathways previously described [50]. Prenatal BPA exposure in a non-human-primate model perturbs early oogenesis and follicle formation in the foetal ovary, illustrating how genotoxic and cytotoxic insults during development can constrain the finite oocyte pool decades before clinical presentation [74]. As in males, human data are starting to parallel the experimental work. Microplastics have now been reported in human ovarian follicular fluid [75], and follicular polyethylene burden has been associated with lower fertilisation rates and with metabolomic shifts in ovarian steroidogenesis and ferroptosis pathways [76].

### 4.3. Assays and Interpretation

These endpoints are captured by a standard genotoxicity toolkit—the comet (single-cell gel-electrophoresis) assay for DNA strand breaks, the micronucleus assay for chromosomal damage, sperm chromatin structure assays, and the immunochemical detection of 8-oxo-dG—applied to gametes, gonadal cells and surrogate lymphocytes [18,71]. Interpreted together with the mechanistic data, they establish that emerging pollutants are not merely endocrine modulators but bona fide reproductive genotoxicants, a distinction with direct implications for the carcinogenic outcomes discussed in Section 5.

## 5. Micro-/Nanoplastics, Chemical Mixtures and the Link to Reproductive Carcinogenesis

### 5.1. Micro- and Nanoplastics as Emerging Pollutants and EDC Vectors

Micro- and nanoplastics deserve particular attention as the newest members of the reproductively active pollutant family. Experimental studies across mammalian and aquatic models show that MNPs impair both male and female reproduction through the same molecular hubs identified above: ROS generation with p38/JNK MAPK activation and Nrf2 depletion, blood–testis-barrier disruption via mTORC1/mTORC2 imbalance, suppression of the LHR/cAMP/PKA/StAR steroidogenic axis, and NLRP3/caspase-1- and TLR4/NOX2-mediated granulosa-cell pyroptosis and ovarian fibrosis [8,42,43,44,46,47,49,70]. Polyethylene-terephthalate and polystyrene particles reduce sperm quality and testosterone and increase spermatogenic-cell apoptosis through oxidative stress and p38 signalling [44,47].

Two findings refine this picture. First, toxicity is strongly size-dependent and cell-type-specific: in a single-cell atlas of the pubertal mouse ovary, only the 100 nm fraction of polystyrene particles was internalised by granulosa cells, and the resulting injury proceeded through cytoskeletal remodelling coupled to epigenetic reprogramming rather than through receptor engagement [50]. Second, effects persist beyond the exposed generation, with F1 and F2 male offspring of perinatally exposed dams showing impaired reproductive development through mechanistically distinct routes [16]. Together these results indicate that bulk-tissue, single-generation designs understate both the specificity and the duration of particulate reproductive toxicity.

MNPs also act as carriers: their large hydrophobic surface adsorbs bisphenols, phthalates and persistent organics, so that co-exposure can amplify combined reproductive toxicity relative to either agent alone [8,9]. Detection of microplastics in human placenta underscores the plausibility of direct human reproductive exposure [7], and particles have since been reported in human semen and ovarian follicular fluid and across a widening range of human tissues and biological fluids [33,72,75].

### 5.2. Chemical Mixtures and Critical Windows

Real-world exposure is never to a single agent but to complex, low-dose mixtures, which may act additively or synergistically and which single-compound risk assessment underestimates [10,15]. Susceptibility is further modulated by developmental timing: in utero and perinatal exposures disrupt germ-cell and gonadal development, peripubertal exposures alter hormonal set-points, and preconception exposures degrade gamete quality [3,74]. Mixture-aware statistical approaches—weighted quantile-sum, quantile g-computation and Bayesian kernel-machine regression—are increasingly required to capture cumulative effects, and non-monotonic dose–response behaviour means that low-dose effects cannot be extrapolated from high-dose testing [10,36].

### 5.3. From Genotoxicity to Reproductive Carcinogenesis

The molecular lesions that impair fertility—receptor hyperstimulation, oxidative DNA damage, epigenetic dysregulation and genomic instability—are also recognised drivers of hormone-dependent and reproductive-tract malignancy [5,14,15]. Structurally diverse EDCs converge on a shared set of oncogenic hubs: ER/AR/GPER-mediated proliferative signalling, AhR activation, PPAR-linked metabolic reprogramming, and ROS-driven DNA damage and apoptosis resistance [15]. BPA and its analogues promote proliferation of ER-positive breast-cancer cells and, through membrane GPER, drive human seminoma-cell proliferation via PKA/PKG signalling [15,40]. Epidemiological and mechanistic syntheses associate EDC exposure with breast, endometrial, ovarian, prostate and thyroid cancers, and a systematic review and meta-analysis links endocrine-disrupting chemicals to elevated risk of testicular germ-cell tumours, including a role for gestational pesticide and organochlorine exposure [14,15]. A meta-analysis of epidemiological studies associates organochlorine pesticides and several PCB congeners with increased breast-cancer risk [77], and mechanistic reviews of endometrial cancer implicate BPA-driven receptor and transcription-factor signalling together with AhR activation by combustion-derived contaminants [78]. Epigenetic mechanisms—altered DNA methylation, histone modification and microRNA expression—are increasingly proposed as the memory by which early exposure is translated into late malignancy [79], and recent syntheses extend the same framework across the gynaecological malignancies as a group [80]. Pesticides contribute a specifically genotoxic component—DNA adducts, strand breaks and chromosomal aberrations arising from oxidative stress—while micro-/nanoplastics exert intrinsic genotoxicity in human cells [15,71]. Thus the reproductive and carcinogenic effects of these pollutants are two outcomes of one underlying molecular toxicology, unfolding over different latencies (Figure 5).

## 6. Alleviation and Mitigation of Emerging-Pollutant Toxicity

Because oxidative stress is a central and shared node of pollutant toxicity, antioxidant and Nrf2-targeted interventions are a rational mitigation strategy at the molecular level. Experimental and translational studies indicate that restoring redox balance—through activation of the Keap1–Nrf2 pathway and supplementation with antioxidants such as N-acetylcysteine, vitamins C and E, melatonin and dietary polyphenols—can attenuate BPA- and microplastic-induced germ-cell apoptosis, lipid peroxidation and DNA damage in reproductive tissues [12,46]. Such approaches remain largely preclinical, but they define a mechanistically grounded avenue for protecting gamete quality in high-exposure settings and a template for biomarker-guided intervention [12].

Molecular mitigation is nonetheless secondary to reducing exposure, which at the individual level is immediately actionable. Environmental history-taking—covering occupational exposures, use of plastics for food storage, pesticide handling and personal-care-product use—can be incorporated into fertility evaluation, and pragmatic preconception counselling (avoiding heating food in plastic, favouring glass or stainless steel, limiting canned and highly packaged foods, improving ventilation and using water filtration where PFAS contamination is suspected) can lower internal dose [11,12]. These measures are supported by intervention data: a scoping review of 58 behavioural, clinical and policy interventions found that most reduced measured bisphenol or phthalate exposure, with policy-level measures producing the largest effects [81], and a randomised controlled trial of a low-plastic diet subsequently confirmed that a short dietary intervention lowers urinary phthalate and bisphenol A concentrations [82]. The route-specific differences set out in Section 2.2 matter here, since the most effective advice differs by class: dietary measures address phthalates, bisphenols and organochlorines, whereas dust and ventilation measures are more relevant to flame retardants and particulates. Within ART, green-IVF practices that minimise clinic-based contaminants extend existing quality and safety standards [11].

At the population level, however, the persistence and mobility of these pollutants make source control and regulation decisive, since individual avoidance cannot address contaminants already distributed through water, food and dust. The molecular evidence argues strongly for class-based rather than substance-by-substance regulation: because BPS and BPF reproduce the receptor and oxidative activity of BPA, and short-chain PFAS retain the persistence of their predecessors, replacing a banned compound with a structural analogue perpetuates the underlying hazard—the phenomenon of regrettable substitution [20]. For the pseudo-persistent pollutants, the regulatory target differs again, since PPCP concentrations in surface water are governed by wastewater treatment capacity rather than by production bans [6]. Reducing the burden of environmentally mediated infertility and reproductive cancer therefore requires harmonised identification and testing criteria, investment in biomonitoring infrastructure, treatment technologies capable of removing pharmaceutical residues, and remediation of persistent contaminants, complemented by the personal and clinical measures above [15,83,84,85].

## 7. Conclusions: Synthesis, Limitations and Research Gaps

Endocrine-disrupting chemicals and related emerging pollutants impair human reproduction through a coherent and largely shared molecular toxicology. Structurally diverse xenobiotics—phthalates, bisphenols, PFAS, pesticides, legacy organochlorines, flame retardants, PPCPs and micro-/nanoplastics—converge on a limited set of mechanisms: disruption of HPG-axis signalling, hormone-receptor interference, steroidogenic-enzyme inhibition, ROS generation with mitochondrial and Keap1–Nrf2 disruption, epigenetic reprogramming, and metabolic–immune crosstalk. These lesions produce measurable cytotoxic and genotoxic damage to gametes and the early embryo, providing a mechanistic foundation for the epidemiological associations with diminished ovarian reserve, prolonged time-to-pregnancy, impaired semen quality and poorer ART outcomes, and—over longer latencies—for the link between these pollutants and reproductive-tract carcinogenesis.

Several limitations of the evidence base should temper these conclusions. The human literature is dominated by cross-sectional studies and by populations recruited from infertility clinics, which constrains causal inference and raises the possibility that associations are strengthened by selection. Exposure assessment for non-persistent compounds frequently rests on single spot urine measurements, which, given urinary half-lives of hours, produces substantial misclassification, generally biasing estimates toward the null. Mechanistic evidence derives predominantly from rodent studies, often at doses above environmental concentrations and by routes that differ from human exposure; extrapolation is complicated further by non-monotonic dose–response behaviour, which means that high-dose findings cannot simply be scaled down. Mixture designs remain rare despite mixture exposure being universal. Umbrella reviews of the wider EDC literature reach a comparable verdict, rating the majority of exposure–outcome associations as being of low or very low certainty [34]. For the newest classes the imbalance is starkest: the mechanistic case for micro-/nanoplastics and for PPCPs rests almost entirely on animal and in vitro work, and where we have described molecular pathways for these pollutants we are describing experimental systems rather than demonstrated human mechanisms.

The gaps that follow are of two kinds. Some that are addressed by this review are: the separation of fertility and carcinogenesis studies, the exclusion of pseudo-persistent pollutants from persistence-based mechanistic frameworks, and the absence of certainty grading that leaves readers unable to weigh the evidence they are given. Others it cannot. There is no human reproductive epidemiology for diclofenac or for most pharmaceutical residues at environmentally relevant concentrations, so the reproductive risk of a very large class of continuously discharged bioactive compounds is presently unquantified. Validated effect biomarkers linking exposure to molecular harm in vivo—of oxidative DNA damage, epigenetic change and receptor activation—do not yet exist in usable form. And no human study has demonstrated the multi-generational transmission shown in rodent models; the recent finding that descendant generations mount compensatory rather than merely attenuated molecular responses [16] indicates that the human studies required would need to be designed around endpoints that existing cohorts do not collect.

Several priorities follow. Mechanistically, the field needs mixture-aware, exposomic and multi-omic studies that capture cumulative and non-monotonic effects and that connect specific molecular initiating events to reproductive and oncogenic outcomes; the cell-type resolution now achievable by single-cell approaches [50] offers a route to the tissue-specific mechanisms that bulk-tissue designs obscure. Micro- and nanoplastics, as both pollutants and vectors, warrant dedicated human mechanistic and biomonitoring research, as do PPCPs, for which environmental occurrence data are far ahead of reproductive epidemiology. Analytically, validated effect biomarkers would enable exposure to be linked to molecular harm in vivo. Therapeutically, Nrf2-targeted and antioxidant strategies deserve rigorous translational evaluation. Recent society-level syntheses of environmental influences on male fertility identify the same priorities and add climate-related heat exposure as an interacting stressor [86]. Finally, the molecular evidence supports a shift toward class-based regulation to prevent regrettable substitution. Elucidating these molecular mechanisms is not only of toxicological interest but a prerequisite for protecting human reproductive health from a growing and pervasive chemical burden.

## Figures and Tables

**Table 1:** Dominant (●) and contributory (○) mechanisms by compound class, with sex-specific reproductive endpoints. HPG, hypothalamic–pituitary–gonadal axis; Rec, nuclear and membrane receptor interference; Ster, steroidogenic-enzyme inhibition; Ox, oxidative stress and mitochondrial dysfunction; Epi, epigenetic reprogramming; Met, metabolic and immune crosstalk. ↓ and ↑ denote a decrease and an increase in the endpoint relative to lower-exposure comparison groups.

Compound Class	Mechanisms	Male Endpoints	Female Endpoints
Phthalates	Rec ● Ster ● Ox ○	↓ testosterone, ↓ sperm concentration and motility, ↑ DNA fragmentation	↓ AMH, ↓ AFC, longer TTP, ↓ oocyte yield and embryo quality
Bisphenols	Rec ● Ox ● Ster ○ Epi ○ HPG ○	↓ motility, abnormal morphology, sperm DNA damage	↓ AMH and AFC, ↓ fertilisation and implantation rates
PFAS	Rec ● Met ● Ox ○ HPG ○	↓ motility, abnormal morphology, altered testosterone	↓ fecundability, ↓ oocyte yield, ↓ live birth
Pesticides	Ox ● Epi ● Rec ○ HPG ○	↓ concentration, motility and morphology (occupational)	Longer TTP, ↑ miscarriage, ↓ ovarian-reserve markers
PCBs/dioxins	Rec ● (AhR, thyroid) Epi ○ HPG ○	↓ semen quality in exposed cohorts	Longer TTP, menstrual irregularity, intergenerational ↓ fecundity
Flame retardants	Rec ● (thyroid) Ox ○	Possible ↓ motility and normal morphology	Longer TTP, ↓ ART success
PPCPs	Rec ● Ster ○ Ox ○	↓ sperm concentration and motility (triclosan, antibiotic mixtures)	↓ AFC, longer TTP (triclosan)
Micro-/nanoplastics	Ox ● Ster ● Epi ● Met ○ HPG ○	Blood–testis-barrier disruption, ↓ testosterone, spermatogenic apoptosis	Granulosa-cell apoptosis, pyroptosis and autophagy; follicular atresia, ↓ oocyte quality

**Table 2:** Phthalate exposure and human reproductive outcomes.

Population	Outcome(s)	Key Mechanistic and Epidemiological Findings	Certainty (GRADE)
Women (general)	Ovarian reserve (AMH, AFC)	Inverse association with DEHP metabolites; StAR/aromatase inhibition and oxidative stress	Moderate
Couples	Time-to-pregnancy	Longer TTP; reduced fecundability	Moderate
Women (ART)	Oocyte yield, embryo quality, live birth	Reduced oocyte yield, poorer embryos, lower implantation/live-birth rates	Moderate
Men	Semen parameters, hormones	Lower sperm concentration/motility; increased DNA fragmentation and aneuploidy; reduced testosterone	Moderate

**Table 3:** Bisphenol exposure and human reproductive outcomes.

Population	Outcome(s)	Key Mechanistic and Epidemiological Findings	Certainty (GRADE)
Women (general)	Ovarian reserve (AMH, AFC)	Higher BPA associated with lower AMH and AFC; ER/GPER signalling, oxidative stress	Moderate
Women (ART)	Oocyte yield, embryo quality, live birth	Fewer mature oocytes; lower fertilisation, implantation and live-birth rates	Moderate
Men	Semen motility/morphology, sperm DNA	Reduced motility and abnormal morphology; sperm DNA damage; concentration inconsistent	Low–Moderate
Substitutes (BPS/BPF)	Cross-class activity	Hormonal/oxidative activity comparable to BPA; limited but concordant human data	Low

**Table 4:** PFAS exposure and human reproductive outcomes.

Population	Outcome(s)	Key Mechanistic and Epidemiological Findings	Certainty (GRADE)
Women (general)	Fecundability, miscarriage	Reduced fecundability; some evidence of increased miscarriage risk	Moderate
Women (ART)	Oocyte yield, embryo quality, live birth	Lower oocyte yield, poorer embryos, reduced live-birth rates	Moderate–High
Men	Semen quality, hormones	Reduced motility, abnormal morphology, altered testosterone	Moderate

**Table 5:** Pesticide exposure and human reproductive outcomes.

Population	Outcome(s)	Key Mechanistic and Epidemiological Findings	Certainty (GRADE)
Women	TTP, miscarriage, ovarian reserve	Longer TTP, higher miscarriage risk, reduced ovarian-reserve markers; lower ART implantation	Moderate
Men (occupational)	Semen quality	Strong association with impaired sperm concentration/motility/morphology; oxidative DNA damage	High
Both sexes	Genotoxic/epigenetic	ROS-driven DNA adducts and strand breaks; altered DNA methylation and miRNA	Moderate

**Table 6:** PCB and dioxin exposure and human reproductive outcomes.

Population	Outcome(s)	Key Mechanistic and Epidemiological Findings	Certainty (GRADE)
Women	TTP, menstrual cyclicity	Longer TTP and irregular cycles in exposed populations; AhR/thyroid disruption	Moderate
Men	Semen quality	Reduced sperm quality in exposed cohorts	Moderate
Couples (ART)	Implantation/live birth	Limited evidence of lower ART success	Low

**Table 7:** Flame-retardant exposure and human reproductive outcomes.

Population	Outcome(s)	Key Mechanistic and Epidemiological Findings	Certainty (GRADE)
Women	TTP, ART outcomes	Longer TTP and reduced ART success; thyroid-hormone disruption	Low–Moderate
Men	Semen quality	Possible reductions in motility and normal morphology	Low

**Table 8:** PPCP exposure and human reproductive outcomes.

Population	Outcome(s)	Key Mechanistic and Epidemiological Findings	Certainty (GRADE)
Women (triclosan)	Ovarian reserve (AFC)	Inverse association with AFC; no association with AMH, FSH or estradiol	Low–Moderate
Women (triclosan)	Time-to-pregnancy	Prolonged TTP in prospective cohort	Low–Moderate
Men (triclosan)	Semen quality, fecundability	Reduced sperm parameters; moderate effect sizes, inconsistent dose–response	Low–Moderate
Men (antibiotics)	Semen parameters	Individual and mixture exposures inversely associated with concentration and motility	Low
Both sexes (diclofenac, NSAIDs)	No human reproductive data	Rodent mixture studies show F1 and F2 effects; no human epidemiology identified	Very low (animal only)

## Data Availability

No new data were created in this review. Data extraction and analysis materials are available from the corresponding author on reasonable request.

## Figure and Table Originality

All figures (Figure 1, Figure 2, Figure 3, Figure 4 and Figure 5) and all tables (Table 1, Table 2, Table 3, Table 4, Table 5, Table 6, Table 7 and Table 8) in this manuscript are original works created by the authors specifically for this review. No figure or table is reproduced, redrawn or adapted in whole or in part from any previously published work, and no copyright permission is therefore required. The figures were produced as original scalable vector graphics (SVGs), authored directly in code by the authors and exported to raster format; no figure-preparation service, template library or online diagramming tool (for example BioRender, Servier Medical Art or similar) was used at any stage, and no third-party publication licence is required. Editable vector source files are available on request. Tables were compiled by the authors from the primary literature cited in each table; the data reported are attributed to the cited studies, but the selection, structure and wording of every table are the authors’ own.

## Abbreviations

AFC	antral follicle count
AhR	aryl-hydrocarbon receptor
AMH	anti-Müllerian hormone
AR	androgen receptor
ART	assisted reproductive technology
BPA/BPS/BPF	bisphenol A/S/F
CYP19A1	aromatase
DEHP	di-(2-ethylhexyl) phthalate
EDC	endocrine-disrupting chemical
ER	estrogen receptor
GnRH	gonadotropin-releasing hormone
GPER	G-protein-coupled estrogen receptor
GPR54	kisspeptin receptor (KISS1R)
HPG	hypothalamic–pituitary–gonadal
3β-/17β-HSD	hydroxysteroid dehydrogenase
LC–MS/MS	liquid chromatography–tandem mass spectrometry
MNP	micro-/nanoplastic
Nrf2/Keap1	nuclear-factor-erythroid-2-related-factor-2/Kelch-like ECH-associated protein 1
8-oxo-dG	8-oxo-7,8-dihydro-2′-deoxyguanosine
PBDE	polybrominated diphenyl ether
PCB	polychlorinated biphenyl
PFAS	per- and polyfluoroalkyl substances
PFOA/PFOS	perfluorooctanoic acid/perfluorooctane sulfonate
PPARγ	peroxisome-proliferator-activated receptor γ
PPCP	pharmaceuticals and personal-care products
ROS	reactive oxygen species
StAR	steroidogenic acute regulatory protein
TTP	time-to-pregnancy
